# Management of Urinary Incontinence in Complete Bladder Duplication by Injection of Bulking Agent at Bladder Neck Level into the Proximal Urethra

**DOI:** 10.1155/2016/6237384

**Published:** 2016-01-21

**Authors:** Reza Khorramirouz, Seyedeh Sanam Ladi Seyedian, Sorena Keihani, Abdol-Mohammad Kajbafzadeh

**Affiliations:** Pediatric Urology Research Center, Department of Pediatric Urology, Children's Hospital Medical Center, Pediatric Center of Excellence, Tehran University of Medical Sciences, No. 62, Dr. Qarib Street, Keshavarz Boulevard, Tehran 1419433151, Iran

## Abstract

Bladder duplication is a rare entity in children. The term encompasses a wide spectrum of anomalies from isolated bladder duplication in coronal or sagittal planes to duplicated bladder exstrophy and associated musculoskeletal and visceral anomalies. Given this wide variability, the treatment of these patients is not standardized. We hereby present a female patient with chief complaint of long-standing urinary incontinence who had complete bladder and urethral duplication and pubic diastasis. The patient was treated with bulking agent injection at the incompetent bladder neck and proximal urethra with resolution of incontinence, obviating the need for extensive surgeries.

## 1. Introduction

Bladder duplication is a rare congenital anomaly traditionally classified as complete or incomplete. In complete forms, two bladders are fully apart in the sagittal or coronal plane and have distinct mucosal lining and muscular walls. Usually each bladder receives the ipsilateral ureter and drains into a distinct urethra [1]. This condition may be associated with multiple congenital anomalies mostly in the urogenital and gastrointestinal tracts and also skeletal system [2].

Since a small number of cases are reported in the literature and also given the wide spectrum of anomalies in these patients, the simple classification of bladder duplication may not be able to explain all the observed variations. Additionally, management of bladder duplication is not standardized and should be individualized according to each patient's condition in order to improve the final outcome. We hereby report a patient with complete bladder and urethral duplication and the management of incontinence in this case using a minimally invasive approach.

## 2. Case Presentation

A 14-year-old girl was referred to our tertiary urology clinic for evaluation of long-standing urinary incontinence and complex genitourinary malformations. She had a history of incontinence noted soon after birth and past surgical history was positive for removal of a red mucosal protrusion from her vaginal introitus after birth but no pathologic report was available. She had experienced multiple relapses and also had undergone surgery for approximation of pubic symphysis at the age of five but urinary incontinence persisted. At presentation she was afebrile with no evidence of urinary tract infection. In physical examination she had two distinct urethras and a vulva with two vaginal openings. The patient was able to void from both urethras but reported incontinency from the right urethra. A thorough neurological examination was unremarkable.

Routine laboratory evaluations including blood urea nitrogen (BUN) and serum creatinine levels were within normal limits. Plain abdominal radiography showed sacral hypoplasia and pubic symphysis diastasis that was previously operated as indicated by metal sutures in place. Ultrasound study reported normal uterus and kidneys but revealed two adjacent cystic masses in pelvis suggestive of duplicated bladder and also showed two distinct vaginal canals separated by a septum and a single normal uterus.

**Figure 1:**
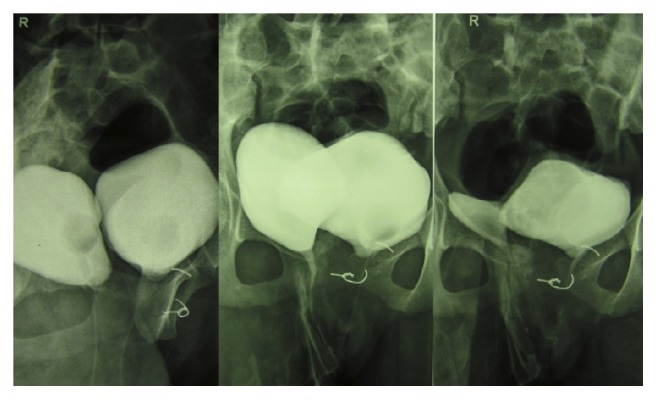
Voiding cystourethrography showing complete bladder duplication with pubic diastasis and sacral hypoplasia.

**Figure 2:**
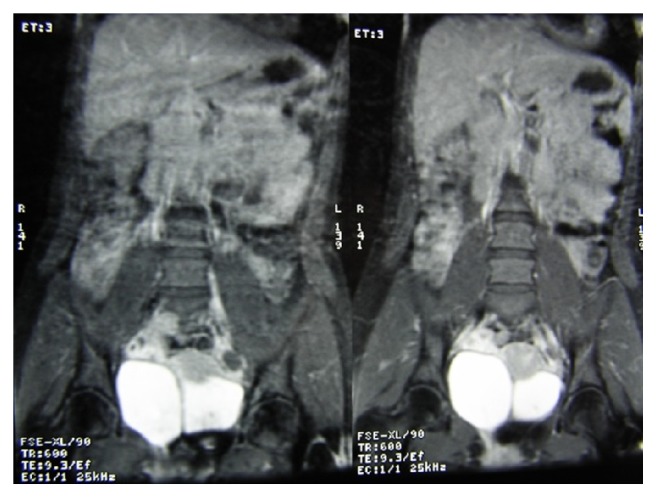
Magnetic resonance urography showing two bladders without communication.

Both urethras were catheterized using 10-Fr. catheters and voiding cystourethrography (VCUG) was performed. VCUG confirmed complete bladder duplication in the sagittal plane with each urethra draining the ipsilateral bladder; no vesicoureteral reflux was noted (Figure 1). Cystoscopy through the right urethra showed the right bladder (volume: 150 cc) with a single horseshoe ureteral orifice and an incompletely developed bladder neck in the distal part. Cystoscopy through the left urethra showed the left bladder (volume: 300 cc) with a normal ureteral orifice and no communication with the right bladder. Vaginoscopy showed two vaginal canals that were distally separated by a septum with confluence near the cervix. Further investigation with magnetic resonance imaging (MRI) and urography (MRU) confirmed presence of a single unicornuate uterus, two separate bladders, and normal kidneys and ureters (Figure 2). Ureters on each side drained into the ipsilateral bladder.

The patient underwent vaginal septum resection. Since the main and only complaint was urinary incontinence, the decision was made to endoscopically correct it without performing an extensive open surgery. Cystoscopy was performed and calcium hydroxyapatite was endoscopically injected into the right bladder neck and proximal urethra as a bulking agent. Urodynamic studies of the bladders after intervention revealed normal patterns in filling and voiding phases (right maximum detrusor pressure: 24 cm H_2_O, end-filling pressure: 6 cm H_2_O; left maximum detrusor pressure: 11 cm H_2_O, end-filling pressure: 2 cm H_2_O). The patient was discharged in good general condition and complete continence without any symptoms. She was dry and satisfied with her condition at 3.5-year follow-up with no urinary tract infection or incontinence. The patient was lost to follow-up afterwards.

## 3. Discussion

In 1961, Abrahamson proposed classifying bladder duplication as either incomplete or complete that occurs in sagittal or coronal planes [1]. Although most duplicated bladders are located side by side in the sagittal plane, fewer are reported in the anteroposterior position (coronal plane) [3]. Interestingly, the latter condition sometimes occurs in association with epispadias or more extensively bladder exstrophy. These findings raise the question whether bladder duplication shares some of the embryologic defects seen in epispadias exstrophy complex (EEC). Lowentritt et al. [4] reviewed more than 815 EEC patients and defined a subgroup of complex EEC variants as “bladder exstrophy with duplication.” This category mostly included anteroposterior bladder duplications in a way that a patch of everted bladder mucosa rests on anterior abdominal wall and the posterior intact bladder lies in pelvis receiving both ureters. In fact, this variant is a shared point in the definitions of bladder exstrophy variants and bladder duplication. Although this definition offers some insight into the common origin of EEC variants and bladder duplication, some patients with duplicated bladders do not have classic findings of exstrophy but have epispadias and also pubic diastasis. For example, our patient had a wide pubic diastasis but both bladders were located in the pelvis without any other sign of bladder exstrophy. Also covered exstrophy with duplicated bladder and visceral sequestration has been previously reported that differs with the classic definition of duplicated exstrophy [5]. Karpathakis et al. [6] also reported an adult male with duplicated bladder in coronal plate and epispadiac duplicated urethra. These findings propose that “Exstrophy-Epispadias Complex with Duplication” encompasses a spectrum of anomalies including conditions like epispadias with bladder duplication, covered bladder exstrophy with duplication, and classic bladder exstrophy with duplication. A duplicated urethra and pubic diastasis may provide invaluable diagnostic clues to this condition.

Regardless of the terminology and the classification used, prompt management of clinical symptoms is the main goal in these patients. Since bladder duplication encompasses a wide range of anomalies (from incomplete to complete bladder duplication and from isolated urinary system anomaly to multiple concomitant anomalies), the clinical significance of this condition largely depends on the associated complaints like incontinence, recurrent infections, and bladder outlet obstruction. However, many patients may be asymptomatic and diagnosed incidentally so treatment is guided by the clinical presentations and should be individualized and targeted to alleviate the symptoms [7]. This may vary from no intervention in asymptomatic cases to intensive surgeries to correct multiple anomalies. In our patient, long-standing urinary incontinence from the right anterior bladder was the sole complaint and continence was achieved with endoscopic injection of bulking agent into the bladder neck sphincter. This approach obviated the need for extensive pelvic surgery to unite the bladders and provided acceptable postsurgical results with full continence. Similarly, Quiroz-Guerrero et al. [8] reported a case of complete duplication of bladder and urethra in the sagittal plane with presence of a vaginal septum and visceral sequestration. Given that both urinary systems had acceptable urodynamic functions with no incontinence, they did not perform surgical intervention and the patient had excellent functional outcome in a 5-year follow-up [8]. Another similarity of the aforementioned patient to ours is the presence of the mucosal protrusion that was surgically removed at an early stage. Although we did not have access to the pathology report of the excised tissue, in two similar cases the histologic evaluation of tissues showed bowel sequestration with intestinal epithelium [5, 8]. In patients with recurrent urinary infection, persistent incontinence, and in those with abnormal function in one or both of the bladders, more invasive surgical approaches like unilateral cystectomy or augmentation cysto-cystoplasty may be preferred [9].

Taken together, the current classification of bladder duplication by Abrahamson [1] does not fully explain the wide variations encountered in reality and a more comprehensive and detailed classification may shed some light on the embryological origins of this anomaly. This is particularly true for patients with lower urinary tract duplication and concomitant signs of bladder exstrophy [10]. The surgical approach in bladder duplication may be individualized and injection of bulking agent into the bladder neck may provide an alternative noninvasive approach.
